# Atrial septal defect occluder combined with stent graft for the management of post-dissection aortic aneurysm previously treated with unsuccessful coils: a case report

**DOI:** 10.1186/s42155-023-00405-7

**Published:** 2023-11-29

**Authors:** Qiqi Wang, Haijun Wei, Chunshui He, Yang Liu

**Affiliations:** Department of Vascular Surgery, Hospital of Chengdu University of Traditional Chinese Medicine, Chengdu, China

**Keywords:** Aortic dissection, Atrial septal defect occluder, False lumen, Thrombosis

## Abstract

**Background:**

Although the candy-plug technique has been reported to be useful for the treatment of post-dissection aortic aneurysm, the stent graft needs be to customized to accommodate the size of vascular occluders.

**Case presentation:**

We present a case of a persistent false lumen successfully treated with endovascular stent-graft and atrial septal defect occluder in a patient with Stanford Type B dissection. A covered stent graft was implanted into the false cavity through a distal rupture, and an atrial septal defect occluder was inserted into the covered stent to seal of the false cavity. Decreased aneurysmal diameter and false lumen thrombosis were noted by CT scan at 6-month follow-up.

**Conclusions:**

Our case showed that combined use of a stent graft and atrial septal defect occluder is safe, technically feasible and effective in sealing of the false lumen in post-dissection aortic aneurysm patients with previously failed false lumen thrombosis.

## Introduction

Post-dissection aortic aneurysm (PDAA) is a serious medical condition associated with significant patient morbidity and mortality. Although open surgery still serves as the first-line choice for managing PDAA, this procedure is invasive and requires high technical demands. On the contrary, endovascular treatments, which is less invasive, has been shown to confer similar favorable patient outcomes as compared with open surgery [1]. Although various endovascular strategies have been reported for PDAA, they can be broadly categorized into the trans-true lumen approaches and trans-false lumen approaches. In particular, although the trans-true lumen fenestrated and branched graft are widely used for the endovascular treatment of juxtarenal and thoracoabdominal aneurysms, this procedure is technically challenging. Embolization of the false lumen at the level of the distal descending aorta in chronic aortic dissection is a less invasive but also effective approach. In recent years, the candy-plug technique, which uses a customized stent graft to seal off the distal false lumen, has been shown to be both technically feasible with encouraging early and late outcomes [2]. In this report, we successfully modified this technique that made midsection tapering down unnecessary by using an atrial septal defect occluder instead to embolize residual arterial flow in the aortic false lumen.

## Case presentation

**Fig. 1:**
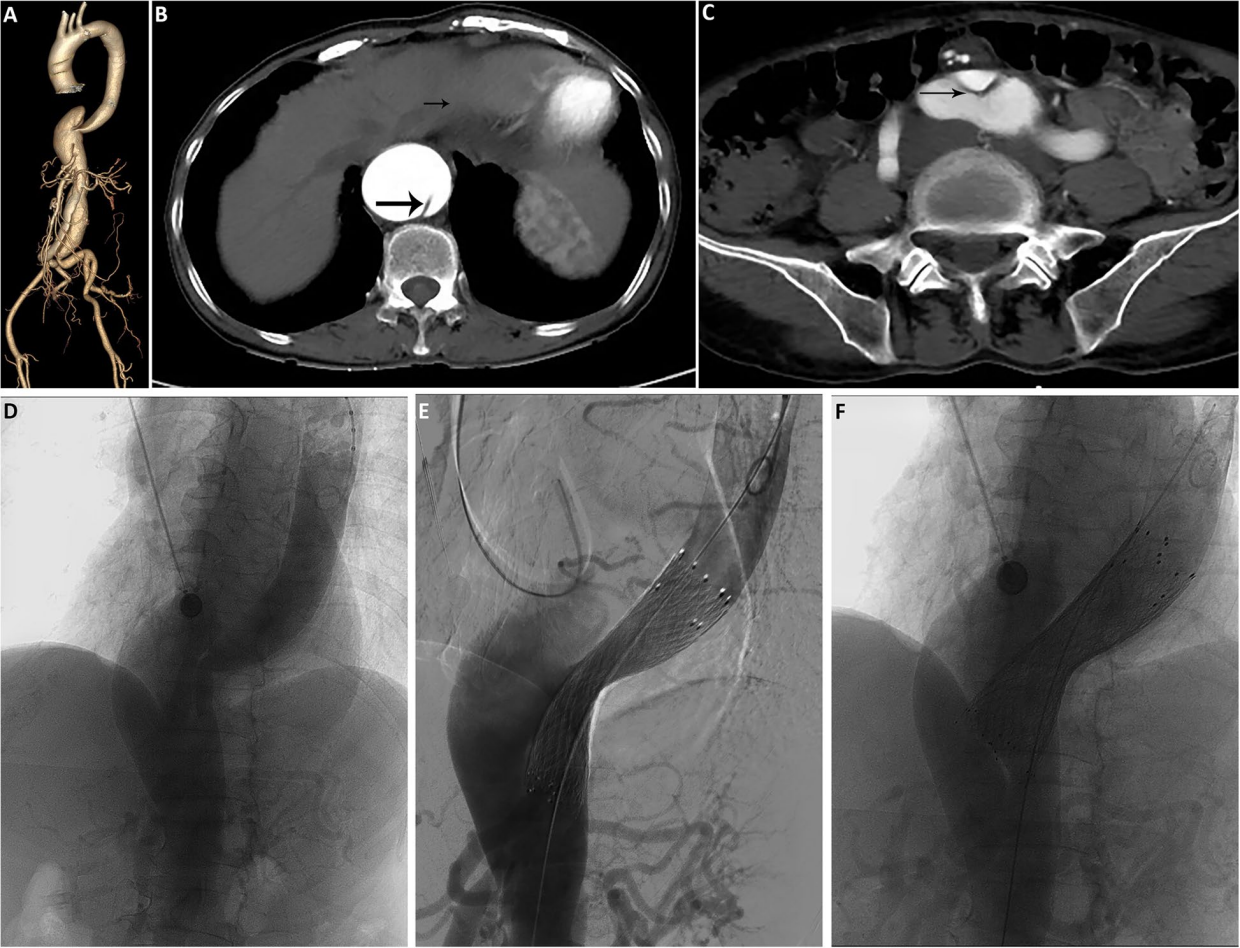
Multi-layer bare metal stents occluded the aortic dissection to reduce blood flow velocity in the false lumen. **A**-**C** Preoperative CTA image of the aorta. Arrow shows the intimal flap of aortic dissection. **D** Preoperative DSA image. **E** Image of a metal bare stent implanted during the procedure. **F** Angiogram on completion of the procedure

An 56-year-old female patient presented with complaints of chest pain on exertion. On admission, the patient was normotensive with a mean arterial pressure of 70-85mmHg and a heart rate of 70 beats/min. A palpable mass was noted by physical examination, which was confirmed to be Stanford type B (De Bakey IIIB) acute aortic dissection by computed tomography angiography (CTA). The primary entry tear was located above the celiac trunk artery, while the distal tear connected to the enlarged pseudoaneurysmal sac was located near the aortic bifurcation (Fig. 1A-C). The patient was managed with the multi-layer bare-metal stent technique, in which 4 bare metal stents (one 24 × 80mm, one 28 × 80mm and two 26 × 80mm, Sinus-XL stent, OptiMed, Ettlingen, Germany) were deployed at the proximal segment of the descending aorta around the primary entry tear. Post-procedural angiography showed significantly reduced blood flow in the false lumen (Fig. 1D-F) and the patient was discharged uneventfully.

**Fig. 2:**
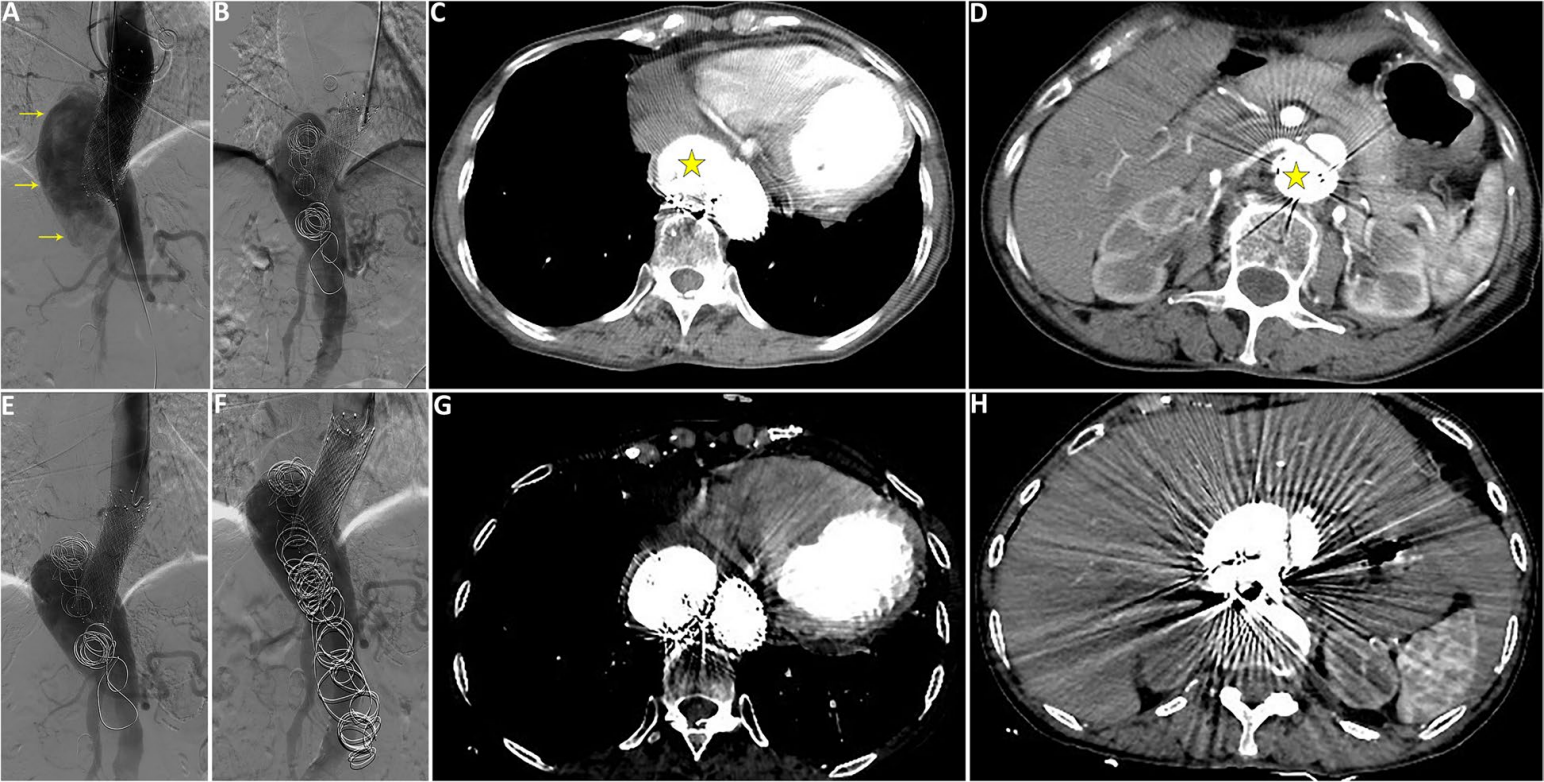
Coil embolization of the false lumen of post-aortic dissection aneurysm. **A** Preoperative DSA image showing rapid flow velocity in the false lumen (yellow arrow); **B** Intraoperative image of spring coil embolization of the false cavity, **C**, **D** CTA image 1 year after coil embolization showing no obvious thrombogenesis in the false lumen (yellow asterisk). **E** Preoperative image of the second coil embolization of the false lumen. **F** Image after the second spring coil embolization of the false cavity. **G**, **H** Six months after the second coil embolization, CTA showed still no obvious thrombus in the false lumen

Six years later, the patient complained of recurrent exertional chest pain, which was attributed to the enlarged descending aorta to more than 60mm with a patent false lumen on CTA. An endovascular procedure was performed to embolize the false lumen by using 3 vascular coils (20 mm*40 cm, Boston Scientific Interlock-35, USA). The angiography on completion revealed significantly reduced backflow in the false lumen and the patient was scheduled follow-up visits on a 6-month interval. Regrettably, subsequent imaging 1 year later showed gradual increase in the diameter of the descending aorta with a patent false lumen (Fig. 2A, B) and no false cavity thrombosis (Fig. 2C, D). Thus, additional 4 vascular coils were delivered again to the false lumen (Fig. 2E-H), since it was the only available material readily available.

**Fig. 3:**
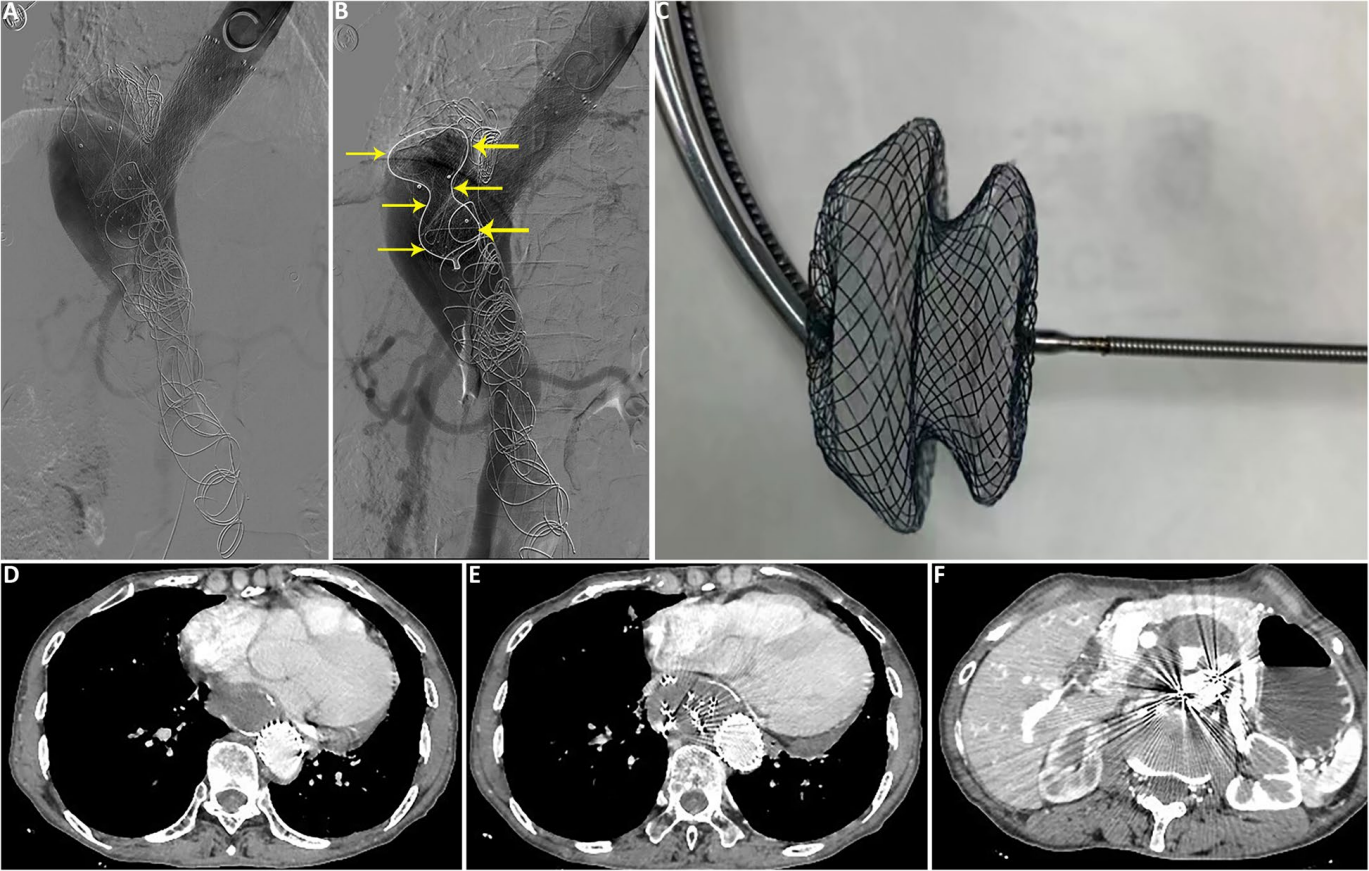
Atrial septal defect occluder combined with covered stents to embolize the false lumen of post-aortic dissection aneurysm. **A** The covered stent was implanted into the false lumen of the aortic dissection through the distal rupture. **B** An atrial septal defect occluder was inserted into the covered stent; **C** Image of an atrial septal defect occluder. **D**-**F** Six months after the procedure, CTA showed obvious thrombus in the false lumen

In the next 2 years of follow-up, CTA scan showed suboptimal false cavity thrombosis and aortic remodeling, and the patient experienced pain that can only be managed with analgesics. Therefore the patient was re-admitted for further treatment. In view of the unfavorable effect of coil embolization in this case, we decided to switch to the candy-plug technique with modifications for false lumen embolization. In brief, instead of tapering the midsection of the stent graft to 10-18mm to accommodate to the size of the vascular plug, an atrial septal defect occluder was used. At the procedure, the stent-graft was installed into the false lumen through the distal re-entry tear, followed by sealing of the distal re-entry tear by release of an atrial septal defect occluder (42–28-38mm, Starway Medicak Technology, Inc.) with a neck length of 4mm and a diameter of 28mm (Fig. 3A-C). Patient’s pain decreased significantly and obvious thrombosis of the false lumen was observed by CTA 6 month post-operatively (Fig. 3D-F).

## Discussion

Endovascular treatment has being increasingly favored in recent years for the treatment of acute type B aortic dissection due to its less invasiveness and quick recovery. Nonetheless, residual false lumen flow due to distal re-entry tears can still be observed in 20%–30% of patients receiving endovascular treatment. Moreover, this persistent false lumen perfusion has been independently associated with poor long-term survival, whereas aortic remodeling after false lumen thrombosis could enhance aortic stability and predict a favorable patient outcome after endovascular aortic repair [3]. False lumen thrombosis in the current case failed due to the backflow of the distal re-entry tear, which resulted in the gradual increase in the diameter of the aorta and persistent symptoms of pain.

Attention to the patency of the branches of the abdominal artery presents as the cardinal consideration in the endovascular management of extensive thoracic and thoracoabdominal aortic aneurysms, as spinal cord ischemia may occur even after the supply of blood vessels such as the celiac axis, superior mesenteric artery and renal arteries were satisfied by endovascular treatment. The patient was first managed with multi-layer bare metal stents in 2014 with satisfying outcomes. In particular, multi-layer bare stent has been shown hemodynamically to reduce blood flow velocity and pressure within the aneurysm vortex, while improving the laminar flow in the main artery and side branch [4]. A meta-analysis with regard to the safety and effectiveness of multi-layer bare metal, however, indicated that the rate of aneurysmal exclusion was only 76.1% and 6.7% patients still need re-intervention [5].

Our case serves as a potential solution for vascular surgeons managing PDAA using the candy-plug technique while faced with unavailability of large vascular occluders. The efficacy of this technique was first systematically analyzed by Rohlffs et al.in 2017, in which the authors demonstrated a technical success rate of 100%, clinical success rate of 94% and aortic remodeling rate of 70% [6]. In the presence of repeated failed attempts to use spring coils to embolize the dissected false lumen, favorable results have been achieved using this technique. Technically, the candy-plug technique involved a custom-made stent and vascular plug. However, the largest-diameter vascular occluder commercially available is 22mm that allows sealing of vessels up to 18mm. Consequently, we opted to replace the original vascular plug with a larger diameter atrial septal defect occluder without narrowing the midsection of the stent graft. The potential risk of true lumen compression following covered stent and candy-plug deployment is not a concern in this patient with bare metal stent implantation.

In recent years, the use of atrial septal defect occluders, which are flexible and self-expanding with a ball-and-socket delivery mechanism that can adapt to the individual anatomy, has been increasingly reported in the literature. Hu and coworkers used an atrial septal defect occluder for the first time to close a distal tear in a patient with type B aortic dissection in 2014 [7]. Subsequently, Petrov et al.reported a case of sealing of the false lumen entry in the ascending aorta after dissection Type A surgical repair with the atrial septal defect occluder [8]. In the largest series of 37 patients with chronic DeBakey IIIb aortic dissection treated with arterial septal occluders, Lu and associates reported complete thrombosis of the false lumen in 91.9% patients [9].

## Conclusion

We believe that the combined use of a stent graft and atrial septal defect occluder is safe, technically feasible and effective in sealing the false lumen in PDAA patients. This strategy can be considered in medical centers with limited experience of branched and fenestrated stent-graft and in patients with previously failed embolization of the false lumen.

## Data Availability

Data sharing is not applicable to this article as no new data were created or analyzed in this study.
